# Should a single growth standard be used to judge the nutritional status of children under age 5 y globally? Debate consensus

**DOI:** 10.1016/j.ajcnut.2024.08.006

**Published:** 2024-09-10

**Authors:** Elaine Borghi, Harshpal Singh Sachdev

**Affiliations:** 1 Department of Nutrition and Food Safety, World Health Organization, Geneva, Switzerland; 2 Senior Consultant Pediatrics and Clinical Epidemiology, Sitaram Bhartia Institute of Science and Research, New Delhi, India

**Keywords:** child growth, nutritional status, global growth standard, contextual growth references, malnutrition

## Abstract

The participants in this debate agreed that: *1*) target-based advocacy is required for ensuring countries’ engagement and political commitments toward reducing child malnutrition, and the tools used for monitoring progress should be accurate and pose no risk of harmful consequences; and *2*) physical growth is not the only dimension of nutritional status to be monitored in clinical and public health practice; anthropometry is thus only one of the diagnostic indicators of nutritional status. Key disagreements included methodological approaches for developing a single growth standard to evaluate nutritional status globally; the relative utility of universal and contextual growth standards for clinical practice and public health; the balance of benefits, harms, and acceptability among stakeholders; and their use as a screening or a definitive tool in individual and public health nutrition. Noteworthy agreements for research priorities included comparison of benefits and harms of using universal compared with contextual growth standards/references and different stakeholders’ perception of expectations from and utility of growth standards.

## Narrative Summary

This debate helped crystallize areas of agreement, refined the key disagreements, and suggested future research priorities for forging an evidence-based consensus on the subject. These proceedings represent the authors’ personal views that may be helpful for structuring the various considerations by stakeholders and national governments to enhance the understanding of child growth and its assessment and role in judging nutritional status globally.

## Points of Agreement

Key points of agreement are summarized in Box 1. The need for target-based advocacy for countries’ engagement and political commitments toward the reduction of child malnutrition across the globe is indisputable. Advocacy needs to be balanced, taking into consideration countries’ priorities, available resources, and contexts. Further, the tools used for monitoring progress should be accurate and not lead to any risk of harmful consequences. It is also agreed that physical growth is not the only dimension of nutritional status to be monitored in clinical and public health practice; anthropometry is thus only one of the diagnostic indicators of nutritional status. When evaluating children in clinical settings, the entire spectrum of anthropometry needs to be considered, namely, stature-for-age, weight-for-age, weight-for-stature or BMI-for-age, and head circumference-for-age. Moreover, both authors agree that, for global monitoring of public health, there are standard definitions of the different indicators such as stunting, wasting, and overweight. However, to avoid misinterpretation and prevent misuse in routine clinical and public health settings, appropriate usage and implications of all growth-related indicators needs to be understood by all stakeholders.

BOX 1 **Points of agreement**

• Target-based advocacy is important for ensuring political commitment and monitoring progress of policies and their implementation for improving the welfare and nutrition of children aged <5 y, particularly the marginalized. Advocacy needs to be balanced, taking into consideration countries’ priorities, available resources, and contexts. The tools used for monitoring progress should be accurate and not lead to any risk of harmful consequences.
• Different dimensions of physical growth in children aged <5 y are used in monitoring tools in clinical and public health practice, including for the evaluation of nutritional status. These dimensions are not necessarily correlated in the same way across different contexts. A similarity in linear-growth (stature-for-age) among different populations does not automatically imply corresponding equivalence in other dimensions such as weight-for-stature, weight-for-age, or head circumference. When evaluating children in clinical settings, the entire spectrum of anthropometry needs to be considered.
• Physical growth is not the only dimension of nutritional status to be monitored in clinical and public health practice; anthropometry is thus only one of the diagnostic indicators of nutritional status.
• For global monitoring of public health, there are standard definitions of the different indicators, such as stunting, wasting, and overweight. However, to avoid misinterpretation and prevent misuse in routine clinical and public health settings, appropriate usage and implications of all growth-related indicators needs to be understood by all stakeholders.

Alt-text: BOX 1

## Continuing Controversies

There remain, however, important points where the authors disagree on the very concept of using one single standard for growth assessment and, consequently, measuring nutritional status of the child. A summary of key points of disagreement is presented in Box 2. These issues revolve around methodological approaches for developing a single growth standard for evaluating nutritional status globally; the relative utility of universal and contextual growth standards for everyday clinical practice, public health monitoring, and actions; the balance of benefits, harms, and acceptability among stakeholders; and the use of growth standards as a screening or a definitive tool for evaluating individual and public health nutrition.

BOX 2 **Key points of disagreement**

**Table:** 

Use of single standard: Yes	Use of single standard: No
*Methodological approaches for developing a single growth standard for evaluating the nutritional status globally*	
Seventeen years after their release, the WHO standards have been thoroughly scrutinized and widely implemented. The WHO standards were developed following a strict protocol, and the sample sizes were more than sufficient to accurately and reliably represent the growth of children globally. The statistical methods used were robust and endorsed by leading statistical experts on the analysis of empirical child growth data. Global growth standards are a powerful and effective tool to assess nutrition status of children from different countries and ethnicities and for underscoring the right for every child to grow to their full potential when their nurturing follows health recommendations and care practices associated with healthy outcomes. Despite the small shifts across growth curves among the 6 sites in the Multicentre Growth Reference Study (MGRS), the growth patterns were highly similar, as expected, because the samples of children were based on similar conditions known to be associated with children’s optimal capacity to grow. Individual intervention effects obviously vary among contexts depending on multiple environment conditions and, even if measurable, should not be considered as thresholds for comparing growth patterns across settings. Naturally, if conditions are unlike those of the MGRS sample, the growth pattern will differ, not only shifting the size but also ultimately altering the curve itself—therefore altering the rate of growth expected for the children living in nonoptimal conditions.	Although they are widely implemented, the WHO standards have not been thoroughly scrutinized externally for their validity. The construction of a single, average growth standard based on populations living under conditions associated with unconstrained growth poses major methodological issues for accurately judging nutritional status globally. When considering the enrollment conditions of children for the construction of the WHO standards, these cannot be automatically presumed to be optimally (that is, neither under- nor over-) nourished. Stringent validity of nutritional status is mandatory, using precise biomarkers and nutrient intakes. Robust evidence is also required to determine the validity of various dimensions (metrics) of the growth of children aged <5 y to accurately and uniformly predict nutritional status across different global contexts. There are significant methodological challenges and controversies in *1*) selecting a sufficiently large and representative sample from an adequate number of different countries to accurately capture global variability; and *2*) The stringency of data merging criterion for pooling different (contextual or country) growth patterns into a single global growth standard, including the latter’s ability to predict nutritional and biological consequences. Illustratively, a tolerance window of ±0.5 SD to conclude “striking similarity” is far too lenient considering that the typical improvements in linear growth with nutritional interventions in low- and middle-income countries, if any, are 0.1 SD to 0.2 SD.
*The relative utility of universal and contextual growth standards for everyday clinical practice and public health monitoring and actions*	
The exact purpose of the WHO growth standards is to create a bar to be reached when breaking the vicious intergenerational cycle of malnutrition. When children’s growth is compared with contextual, local references, one misses the important growth characteristics of these children as compared to the optimal growth depicted by children living under conditions associated with unconstrained growth. Parents want their children to reach their full potential and not to accept what is typical in their environment. The use of a single, appropriate global growth standard is essential to accurately describe how a population of children falls short of achieving optimal growth and development, thereby calling attention to the additional efforts needed and putting political pressure on decision makers to invest on behalf of children. The use of local growth references causes confusion and lowers expectations, inhibiting aspirations and action to achieve optimal growth and development for children. Growth standards are crucial for accurately describing and comparing how populations of children are growing.	A single growth standard to judge the nutritional status of children aged <5 y globally has obvious advantages of simplifying comparisons, clinical practice, and policy while sending an impactful message of global equity. However, these advantages should never be achieved at the cost of substantial misclassification, with potentially harmful implications, during practical usage. In routine clinical practice, contextual growth references predict neonatal morbidities, pathological short stature, macrocephaly, and cardiometabolic risk factors better than the WHO standards. Child body composition also varies contextually, with greater adiposity, despite comparable weights, in south Asian populations. There are undesirable consequences of assessing nutritional adequacy from a single growth standard for public health discourse and policy, especially for low- and middle-income countries. The advocacy machinery tends to portray national child undersize statistics as a burden of “hunger” or “starvation.” Inevitably, this is sensationalized by the lay media. The consequent misdirected shaming creates intense political pressures, which demand inappropriate feeding responses that amplify the use of food or nutrient-product based supplementation, which can escalate the ongoing increase in overnutrition burden. Conversely, in high-income countries, overnutrition tends to be underestimated with its own consequences.
*The balance of benefits, harms, and acceptability among stakeholders*	
The use of a single, global growth standard to assess the nutrition status of children from different countries and ethnicities is a powerful and effective tool for underscoring the right of every child to grow to their full potential when their nurturing follows health recommendations and care practices associated with healthy outcomes. The WHO Child Growth Standards represent the best description of optimal physiologic growth and should be applied to all children everywhere, regardless of ethnicity, socioeconomic status, and type of feeding.	The balance of benefits and harms does not favor the use of a single growth standard. The major concerns for the mainstream and underprivileged populations relate to misclassification errors resulting in misdirected stigmatization, inappropriate clinical and public health responses, and use of flawed indicators for monitoring nutrition-related SDGs. Parents also want to know how their children are growing, given their context (conditional performance), and if improvement is required or possible. Setting unreasonable targets creates the despairing and populist impression, often with kneejerk remedies, that the achievement of optimal growth is only for affluent and privileged inhabitants.
*The use of growth standards as a screening or a definitive tool for evaluating individual and public health nutrition*	
Anthropometry has an important advantage over other nutritional metrics. Biochemical and clinical metrics are useful only at the extremes of malnutrition, while body measurements are informative over the full spectrum. This is important for early detection of malnutrition. Moreover, anthropometric metrics provide the best noninvasive indicators of when a child is not reaching their full potential growth, largely but not only due to living in a suboptimal environment for their wellbeing. Using global standards based on expected optimal growth to compare groups of children does not preclude or replace other diagnostics tools during routine clinical visits where practitioner finds necessary.	The use of contextual growth references, while admittedly not the perfect solution, will result in a better fit for everyday practice and policy. Growth standards are only a screening tool to reach a clinical judgment, which needs support from other accurate biomarkers to achieve precision in individual and public health nutrition.

SDG: Sustainable Development Goals.

Alt-text: BOX 2

## Research Agenda to Resolve Debate

Addressing gaps in the currently available evidence could improve our understanding and meaningfully inform the choice of using a global or a contextual anthropometric standard for everyday clinical practice and public health actions. Both authors identified such research items based on their own viewpoints, which did not intersect completely for obvious reasons, as depicted in Box 3. The noteworthy agreements include evidence generation for: *1*) comparing benefits and harms of using universal compared with contextual growth standards/references in routine clinical practice and public health policy, and *2*) different stakeholders’ perception of expectations from and utility of growth standards.

BOX 3 **Research agenda**

**Table:** 

Areas of agreement
• Pooled analysis of available datasets from different contexts for comparing the benefits and harms of using universal compared with contextual growth standards/references used in routine clinical practice and public health policy.
• In-depth evaluation of different stakeholders’ perception of expectations from and utility of growth standards in their lives and for routine clinical practice and public health policy.
• Use of the WHO child growth velocities in specific contexts to protect children from growth failure as well as risk of obesity later in life.
• The MGRS study could be used to explore other anthropometric indicators to cover the current gaps in noninvasive diagnostics for malnutrition to complement the existing ones. This is specifically important for children aged 2 to 5 y, where risk of obesity later in life is higher as the growth rate slows down.
• Development of consensual evidence-based guidance for monitoring growth of preterm or small or large-for-gestational-age infants.
• Innovative ways of depiction and evaluation of growth trajectories, in addition to growth charts for children aged <5 y.
*Additional research priorities by NO (HSS)*
• The underlying philosophy, principles, and methods of creating growth standards need considerable re-examination in the current era, followed by wider stakeholder involvement and possible revision, including from the following perspectives: *1*) ability to predict important biological consequences; and *2*) the stringency required for criteria to allow the merging of different (contextual) growth patterns into a single global growth standard, including the ability to predict biological consequences.
• Secondary analysis of the available WHO-MGRS data to quantify the variability in other physical growth dimensions, such as weight-for-stature, weight-for-age, or head circumference.
• Prospective studies designed to adequately capture global representation and variability to determine contemporary growth patterns and their associations with the biological construct of nutritional status and other functional outcomes.

Alt-text: BOX 3

## Acknowledgments

EB thanks Mercedes de Onis for her legacy on evidence-building in the field, which supported this piece, and to Francesco Branca, Edward Frongillo, and Richard Kumapley for their feedback on earlier drafts of this manuscript. HSS is grateful to Anura V. Kurpad for his constructive feedback on earlier drafts of this manuscript.

## Author contributions

Both authors contributed equally to this article and read and approved the final manuscript.

### Conflict of interest

EB works for the World Health Organization , which recommends the global Child Growth Standards for all countries. HSS declares no conflicts of interest.

## Funding

The authors reported no funding received for this study

## Disclaimer

This article series is designed as an Oxford-style debate. As such, participants are required to argue pro- and con- positions, even when that opinion may differ from their own. The views expressed in this debate do not necessarily reflect the opinion of the participants, *The American Journal of Clinical Nutrition*, or the American Society for Nutrition.

